# Hydrogen sulfide modulates chromatin remodeling and inflammatory mediator production in response to endotoxin, but does not play a role in the development of endotoxin tolerance

**DOI:** 10.1186/s12950-016-0119-2

**Published:** 2016-04-01

**Authors:** Ester C. S. Rios, Francisco G. Soriano, Gabor Olah, Domokos Gerö, Bartosz Szczesny, Csaba Szabo

**Affiliations:** Department of Emergency Medicine, Universidade de São Paulo Medical School, São Paulo, Brazil; Department of Anesthesiology, University of Texas Medical Branch, 601 Harborside Drive, Building 21, Room 4.202D, Galveston, TX 77555-1102 USA

**Keywords:** Hydrogen sulfide, Endotoxin, Cytokines, Macrophages, Tolerance

## Abstract

**Background:**

Pretreatment with low doses of LPS (lipopolysaccharide, bacterial endotoxin) reduces the pro-inflammatory response to a subsequent higher LPS dose, a phenomenon known as endotoxin tolerance. Moreover, hydrogen sulfide (H_2_S), an endogenous gaseous mediator (gasotransmitter) can exert anti-inflammatory effects. Here we investigated the potential role of H_2_S in the development of LPS tolerance. THP1 differentiated macrophages were pretreated with the H_2_S donor NaHS (1 mM) or the H_2_S biosynthesis inhibitor aminooxyacetic acid (AOAA, 1 mM).

**Methods:**

To induce tolerance, cells were treated with a low concentration of LPS (0.5 μg/ml) for 4 or 24 h, and then treated with a high concentration of LPS (1 μg/ml) for 4 h or 24 h. In in vivo studies, male wild-type and CSE^-/-^ mice were randomized to the following groups: Control (vehicle); Endotoxemic saline for 3 days before the induction of endotoxemia with 10 mg/kg LPS) mg/kg; Tolerant (LPS at 1 mg/kg for 3 days, followed LPS at 10 mg/kg). Animals were sacrificed after 4 or 12 h; plasma IL-6 and TNF-α levels were measured. Changes in histone H3 and H4 acetylation were analyzed by Western blotting.

**Results:**

LPS tolerance decreased pro-inflammatory cytokine production. AOAA did not affect the effect of tolerance on reducing cytokine production. Treatment of the cells with the H_2_S donor reduced cytokine production. Induction of the tolerance increased the acetylation of H3; AOAA reduced histone acetylation. H_2_S donation increased histone acetylation. Tolerance did not affect the responses to H_2_S with respect to histone acetylation.

**Conclusions:**

In conclusion, both LPS tolerance and H_2_S donation decrease LPS-induced cytokine production in vitro and modulate histone acetylation. However, endogenous, CSE-derived H_2_S does not appear to play a significant role in the development of LPS tolerance.

## Background

Sepsis, a systemic inflammation caused by pathogens, remains a significant clinical problem [1, 2]. Amongst a multitude of pathophysiological events affecting the cardiovascular and immune system during sepsis, the outcome of sepsis is significantly affected by the severity of vascular disturbances, compromising oxygen delivery to the tissues, contributing to the development of multiple organ dysfunction. In the early phase of sepsis and septic shock there is an intense release of pro inflammatory mediators that can promote tissue injury and multiple organ dysfunction [3–7].

The induction of tolerance to lipopolysaccharide (LPS) has been investigated for several decades as a potential therapeutic approach for sepsis [8–18]. LPS tolerance downregulates the inflammatory response in septic shock while also increasing the ability of a host to eliminate the pathogens; it exerts protective effects in several models of sepsis and polymicrobial infection [14–18]. Inflammatory gene silencing resulting from tolerance can persist for days to weeks [8–10]. The mechanisms involved in LPS tolerance include toll-like receptor desensitization as well as the suppression of the inflammatory signaling pathways that regulate the production of anti-inflammatory cytokines, at least in part via epigenetic changes [11]. Chromatin remodeling during the period of LPS tolerance development modifies gene transcription profile and regulates or silences a host of genes in response to a subsequent challenge [12, 13]. LPS tolerance also modulates the production of reactive oxygen and nitrogen species (ROS/RNS) [16].

Hydrogen sulfide (H_2_S) emerges as a novel gaseous mediator and signaling molecule, with multiple roles in health and disease [19]. Among other pathways, H_2_S regulates the activation of ERK and p38 MAP kinase, modulates cell proliferation and regulates oxidant-induced cell death [19–25]. Here we investigated effect of modulation of H_2_S homeostasis (by H_2_S donation or inhibition of endogenous H_2_S generation) in in vitro/vivo models of LPS tolerance and endotoxemia.

## Methods

### Macrophage culture and differentiation

THP1 monocytes obtained from ATCC were differentiated into macrophage using 100 nM phorbol myristate acetate (PMA) for 5 h in RPMI 1640 supplemented with 2 mM L-glutamine, 100 U/ml penicillin, 100 μg/ml streptomycin and 10 % fetal bovine serum (FBS) (Sigma). Ultrapure Escherichia coli 0111:B4 LPS free of lipoproteins was obtained from Invitrogen (San Diego, CA).

### In vitro model of tolerance and sepsis

**Fig. 1:**
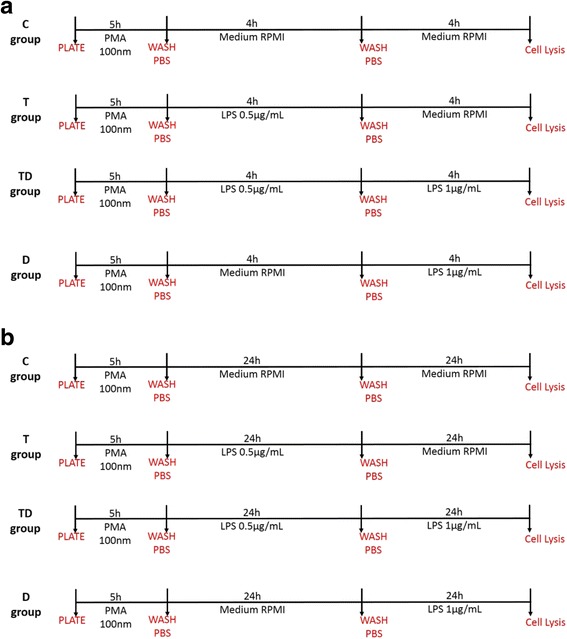
In vitro experimental protocols of LPS tolerance used in the current study

**Fig. 2:**
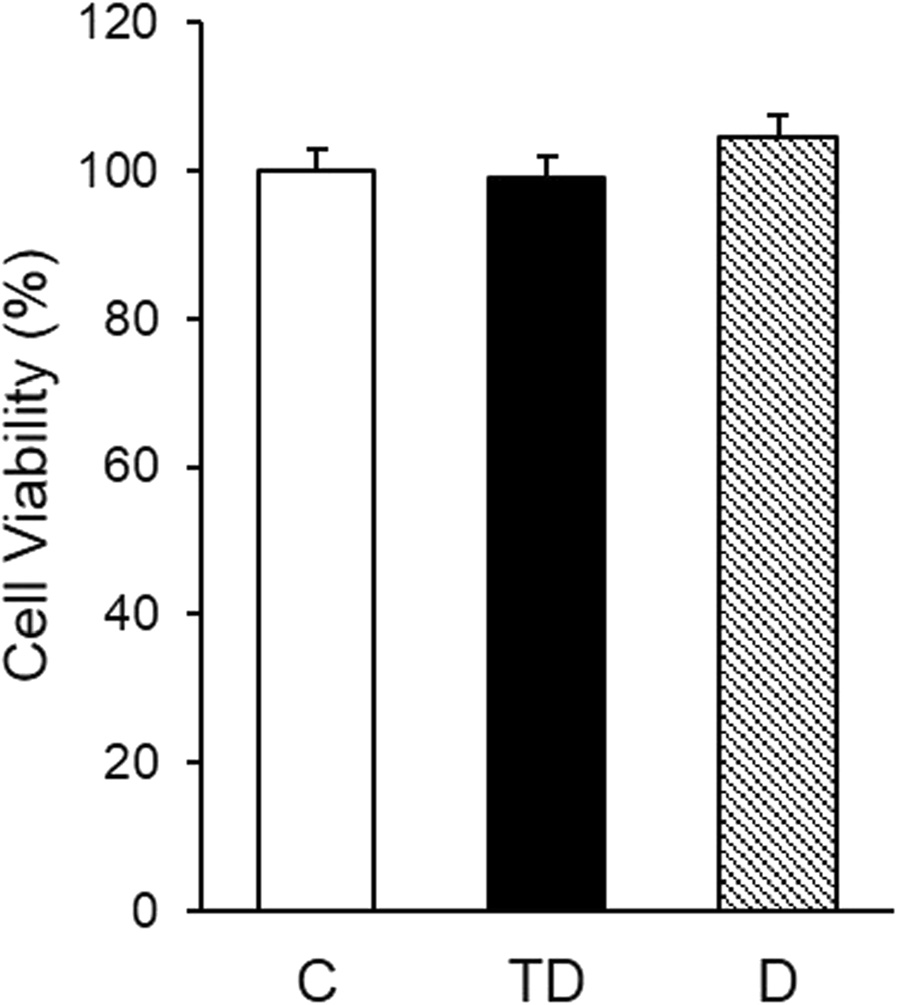
Cell viability in an in vitro model of tolerance and sepsis. Macrophages were stimulated either with a single dose of LPS 0.5 μg/ml followed by a higher dose of LPS (1 μg/ml) (TD group) or directly with LPS (1 μg/ml) (D group). The MTT assay was performed in the experimental design described in the Fig. 1b (48 h after the start of the experiment)

THP1 cells were plated in 22 mm tissue culture dishes (2 × 10^6^ cells/dish). In the first experimental design, four groups of cells were studied (Fig. 1a). Group “C” (i.e. “Control”) was designated as the control group that was maintained with medium and received vehicle only. Group “T” (i.e. “Tolerance”) received a single and low concentration of LPS at 0.5 μg/ml for 4 h, followed by washout, and further incubation for 4 h. Group “TD” (i.e. “Tolerance + Direct Challenge”) received the same low concentration of LPS (0.5 μg/ml) as the “T” group, followed, at 4 h, by a higher concentration of LPS (1 μg/ml) for an additional 4-h period. Group “D” (i.e. “Direct Challenge”) did not receive the low concentration of LPS; instead it received vehicle at the beginning of the experiment, but it received the higher concentration of LPS (1 μg/ml) 4 h later. Culture supernatant was collected 4 h after the challenge with the higher concentration of LPS (1 μg/ml, i.e. 8 h after the start of the experiment). In the next set of experimental design (Fig. 1b), a similar approach was used, but the time period for both the first, tolerizing concentration (0.5 μg/ml) and for the second, higher (“challenge”) concentration (1 μg/ml) was extended to 24 h. In these experiments, culture supernatant was collected 24 h after the challenge with the higher concentration of LPS (1 μg/ml, i.e. 48 h after the start of the experiment). Cell viability was not effected under these experimental conditions (Fig. 2).

**Fig. 3:**
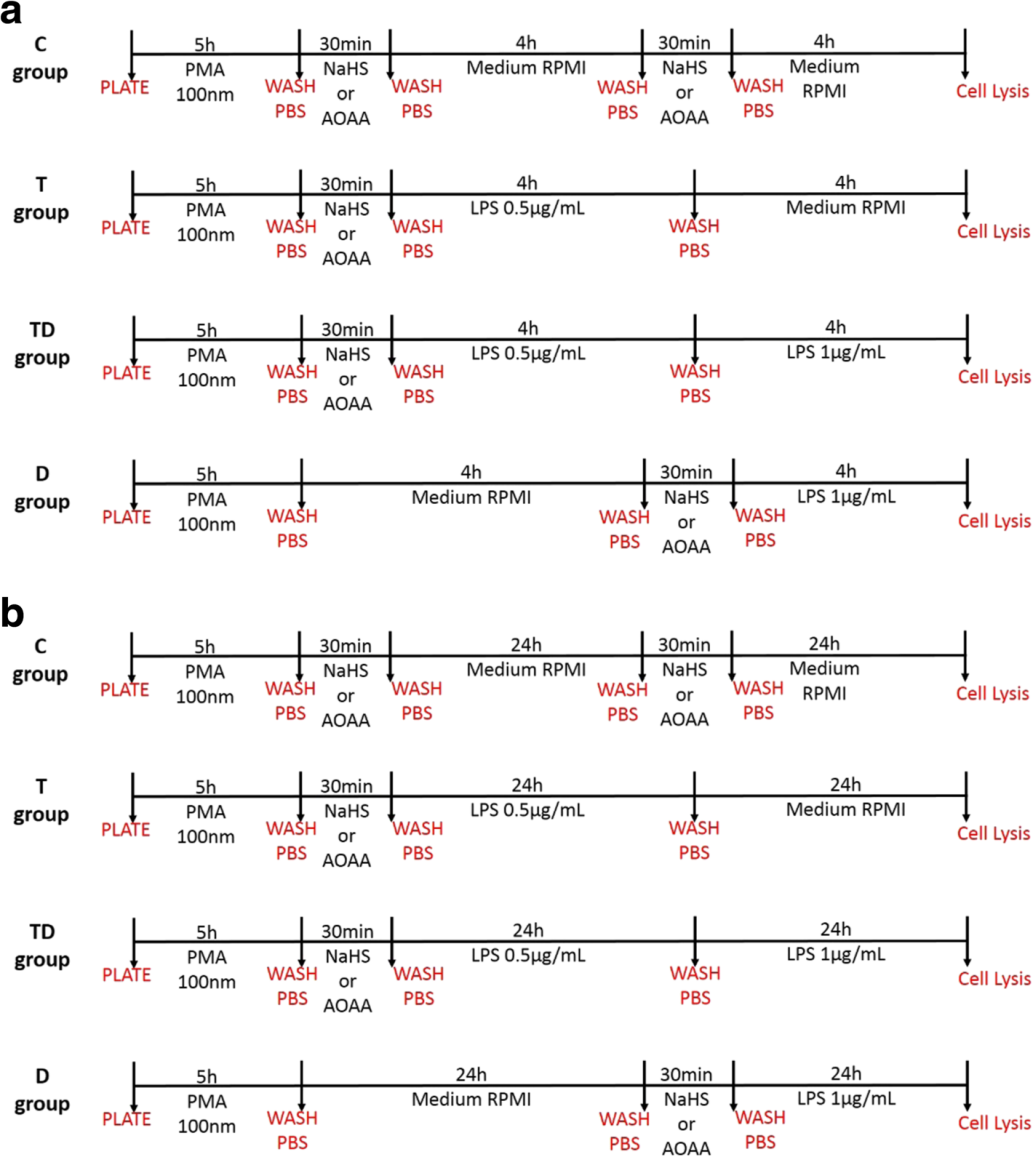
Scheme showing the administration of AOAA and NaHS in the in vitro protocol. Part (**a**) depicts the shorter experimental design (4 h of low concentration of LPS exposure, followed by 4 h of high concentration of LPS exposure, followed by the collection of culture supernatant at 8 h) and part (**b**) depicts the longer experimental design (24 h of low concentration of LPS exposure, followed by 24 h of high concentration of LPS exposure, followed by the collection of culture supernatant at 48 h)

To study the effect of the H_2_S biosynthesis inhibitor aminooxyacetic acid (AOAA) or the H_2_S donor NaHS, the experimental design shown in Fig. 3a and b was employed. The Control group (Group “C”) received 2 sets of treatment (each times 30 min) with the H_2_S modulators (each time, the 30 min exposure was followed by wash-outs, prior to the subsequent application of LPS). Since our goal was to understand the effect of H_2_S modulators on the development of LPS tolerance, the groups that received the low (tolerizing) exposure to LPS (Groups “T” and “TD”) received 30 min of treatment with either NaHS or AOAA *before* the low concentration of LPS. On the other hand, for the group designated to serve as the “Direct Challenge” group (“DC”) by exposing it to the higher concentration of LPS (group “D”) the exposure to NaHS or AOAA was applied 30 min prior to this very stimulus. This experimental design was employed both in the shorter experimental design (4 h of low concentration of LPS exposure, followed by 4 h of high concentration of LPS exposure, followed by the collection of culture supernatant at 8 h) (Fig. 3a) and in the longer experimental design (24 h of low concentration of LPS exposure, followed by 24 h of high concentration of LPS exposure, followed by the collection of culture supernatant at 48 h) (Fig. 3b).

### In vivo model of tolerance and endotoxemia

All procedures were performed in accordance to the Guide for the Care and Use of Laboratory Animals published by the US National Institutes of Health and were was approved by UTMB’s IACUC. Animals were anesthetized (i.p) with a mixture of ketamine (80 mg/kg) and xylazine (10 mg/kg). Male C57bl/6 wild-type mice or cystathionine γ-lyase (CSE)-deficient mice (a kind gift of Dr. Solomon Snyder, Johns Hopkins University, Baltimore, MD) were randomized in the following groups: Group C (control group) - no treatment; Group D (directly challenged/endotoxemic) – received 0.1 ml normal saline i.p. during 3 days before the induction of endotoxemia (LPS 10 mg/kg); Group TD (tolerant + endotoxemic)—animals received LPS 1 mg/kg i.p. during first 3 days before the induction of endotoxemia (LPS 10 mg/kg). 2 ml lactated Ringer’s solution alone i.p. was administered immediately after endotoxemia induction. 4 or 12 h after endotoxemia induction, animals were sacrificed and plasma collected.

### Cell viability

To estimate cell viability of the in vitro model of tolerance described before 3-(4,5-dimethyl-2-thiazolyl)- 2,5-diphenyl-2H-tetrazolium bromide (MTT) was added to the cells at a final concentration of 0.5 mg/ml and cultured at 37 °C for 1 h. Cells were washed with PBS and the formazan dye was dissolved in isopropanol. The amount of converted formazan dye was measured at 570 nm with a background measurement at 690 nm on spectrophotometer (Tecan Genius, Salzburg, Austria). Viable cell count was calculated as a percent of control cells.

### Western blot analysis

THP-1 cells lysed in RIPA buffer and sonicated (3 times of 10 s). The supernatants were preserved and protein concentration was determined by BCA (BioRad). 25 μg cell extract was resuspended in equal volume of loading buffer (20 mM Tris-HCl, pH 6.8; 2 % SDS; 10 % glycerol; 6 M Urea 2 %; 15 % β-mercaptoethanol; urea 6 and 0.01 % bromophenol blue), boiled for 2 min and electrophoresed on 8–12 % SDS-polyacrylamide gels. After electrophoretic separation, proteins were transferred to PDF membranes. Membranes were blocked with T20 Starting Buffer (Thermo Scientific) for 1 h. Following primary antibodies at the dilution 1:1,000 were used: rabbit acetylated histone H3 at N-terminal tail (Millipore 06-599), anti-histone H4 acetylated at lysine 5/8/12/16 (Millipore 06-866), HRP conjugated β-actin (Santa Cruz Biotechnology). The primary antibodies were incubated overnight at 4 °C the membranes were washed twice in TBST. A secondary horseradish-peroxidase-conjugated antibody (goat anti-rabbit, Cell Signaling) was then applied at a dilution of 1:5000 for 1 h. Over a 30-min period, the blots were washed twice in TBST. Signal was obtained using Super Signal Detection Kit (Pierce, Rockford, IL, USA). Band intensity was quantified using Genetools (Syngene, Synoptics Ltd., USA) and normalized to β-actin.

### Measurement of cytokine production by THP-1 cells

Cell culture medium and plasma samples were collected to measure TNF-α and IL-6 by ELISA according to manufacturer’s instructions (R&D Technologies, USA).

### Statistical analysis

All values were expressed as mean ± standard error of the mean (SEM) from 5 or 6 repetitions per group for the cell culture and animal studies. Statistical analyses were performed using GraphPad InStat Software. Comparisons among experimental groups were performed by analysis of variance ONE-WAY ANOVA and Tukey’s test was used as post hoc test to compare individual groups. A p-value less than 0.05 were considered significant.

## Results

### H_2_S modulates the production of IL-6 and TNF-α in a THP-1 model of LPS tolerance

**Fig. 4:**
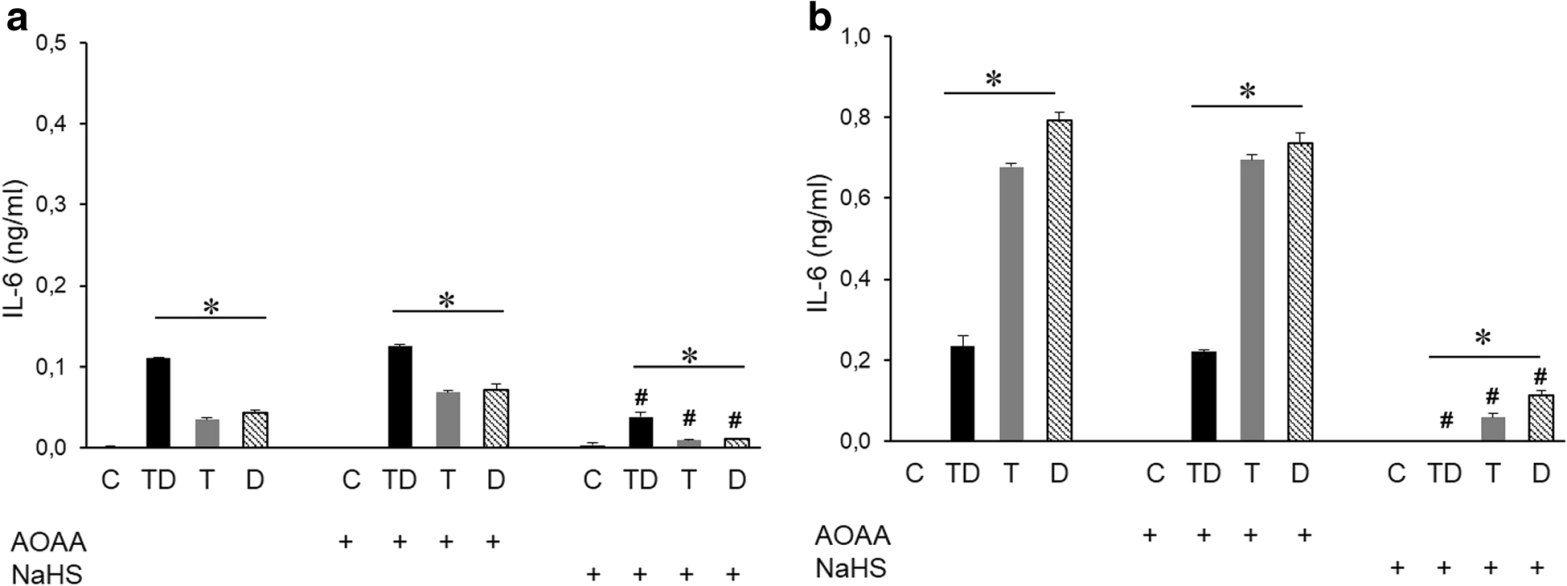
H_2_S modulates the production of IL-6 in THP-1 cells treated with LPS. Macrophages were stimulated either with a single dose of LPS 0.5 μg/ml (T group), a single dose of LPS 0.5 μg/ml followed by a higher dose of LPS (1 μg/ml) (TD group) or directly with LPS (1 μg/ml) (D group). IL-6 concentration in the culture medium was measured after 4 h (**a**) or 24 h (**b**) after the final LPS treatment. The H_2_S biosynthesis inhibitor AOAA and the H_2_S donor were both applied at 1 mM. *p < 0.05 TD vs. D groups; #*p* < 0.05 shows the inhibitory effect of NaHS, compared to the respective group that did not receive the H_2_S donor

**Fig. 5:**
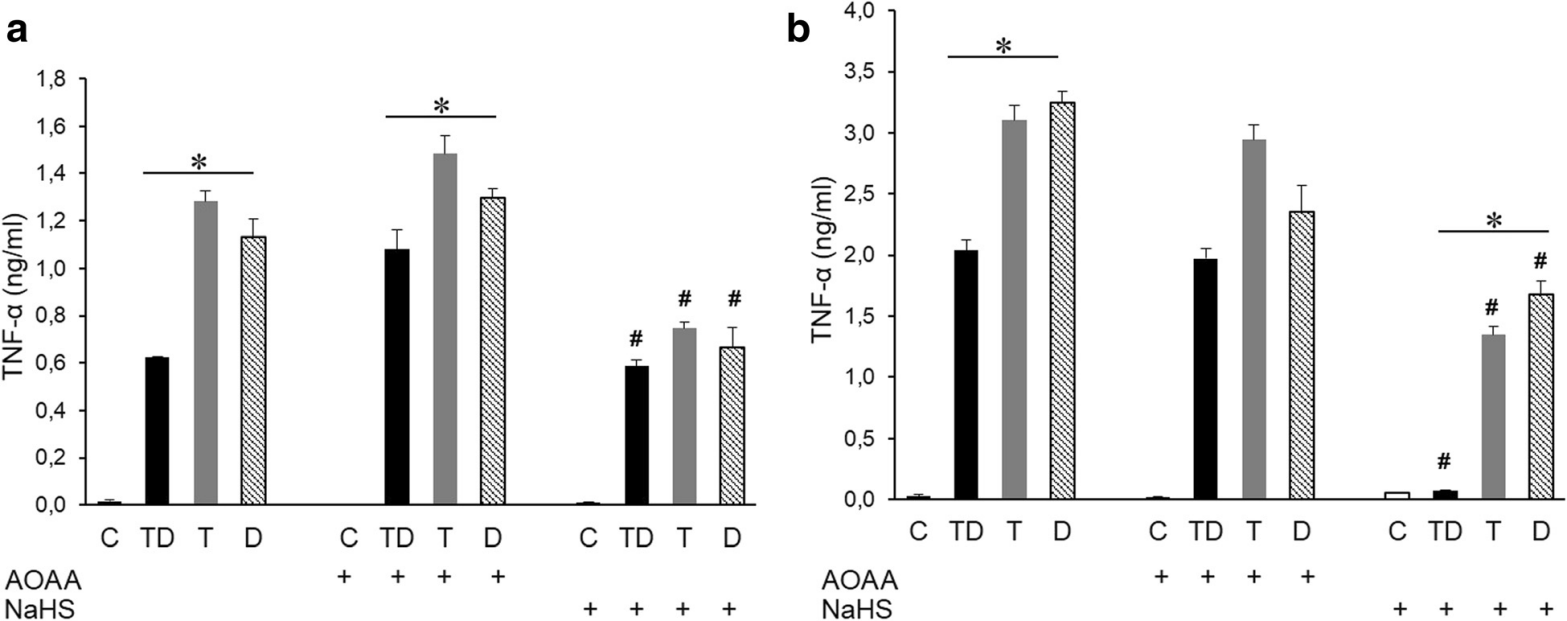
H_2_S modulates expression of TNF-α in THP-1 cells treated with LPS. Macrophages were stimulated either with a single dose of LPS 0.5 μg/ml (T group), a single dose of LPS 0.5 μg/ml followed by a higher dose of LPS (1 μg/ml) (TD group) or directly with LPS (1 μg/ml) (D group). TNF-α concentration in the culture medium was measured after 4 h (**a**) or 24 h (**b**) after the final LPS treatment. The H_2_S biosynthesis inhibitor AOAA and the H_2_S donor were both applied at 1 mM. *p < 0.05 TD vs. D groups; #*p* < 0.05 shows the inhibitory effect of NaHS, compared to the respective group that did not receive the H_2_S donor

We first investigated the amount of IL-6 and TNF-α released into the medium of cultured THP-1 cells treated with various combination of LPS (Figs. 4 and 5). Cells exposed initially to low concentration of LPS followed by high concentration of LPS (Tolerance and Direct Challenge: Group TD) produced significant less IL-6 (Fig. 4b) and TNF-α (Fig. 5b) than the amount of the cytokines produced in the Direct Challenge Group (Group D), validating the development of tolerance in our experimental protocol. Similarly, a pattern of tolerance was noted for TNF-α production in the shorter exposure protocol (shown in Fig. 1a) involving exposure to 4 h of the lower concentration of LPS, followed by 4 h of the higher concentration of LPS (Fig. 5a). Surprisingly, at the same time, in this shorter tolerization/exposure protocol, when evaluating IL-6 production, a pattern of additive cytokine production was seen: the amount of cytokines produced in the TD group was higher than the cytokines produced either by the T group or the D group (Fig. 4a).

Next, we investigated effect of inhibition of endogenous H_2_S generation (using AOAA) or the effect H_2_S donation (using NaHS) on the responses characterized in the prior section. For H_2_S biosynthesis inhibition AOAA was selected, because it is an inhibitor of two major H_2_S generating enzymes: cystathionine-β-synthase (CBS) and cystathionine-γ-lyase (CSE) [26]. We did not observe any significant effect of AOAA on the production of IL-6 and TNF-α, either in the 4 + 4 and 24 + 24 h protocols (Figs. 4 and 5). However, pretreatment of the cells with NaHS significantly reduced the effect of LPS in all experimental groups (Figs. 4 and 5). The most pronounced effect of NaHS was noted on the production of IL-6 and TNF-α at 24 h in Group “T” (Figs. 4b and 5b) suggesting that the inhibitory effect of H_2_S on cytokine production is most pronounced when the longest time is given to exert its effect (in this case, cytokines were measured at 48 h relative to the exposure to NaHS and to 0.5 μg/ml LPS, as compared to the other groups, where the measurements of cytokines were conducted at 24 h after exposure to NaHS and LPS). One may also describe the observed effect of NaHS as follows: in the 48-h protocol it enhanced the tolerance-inducing effect of LPS on TNF-α and IL-6 production.

### H_2_S modulates acetylation of histones 3 and 4 in a THP-1 model of LPS tolerance

**Fig. 6:**
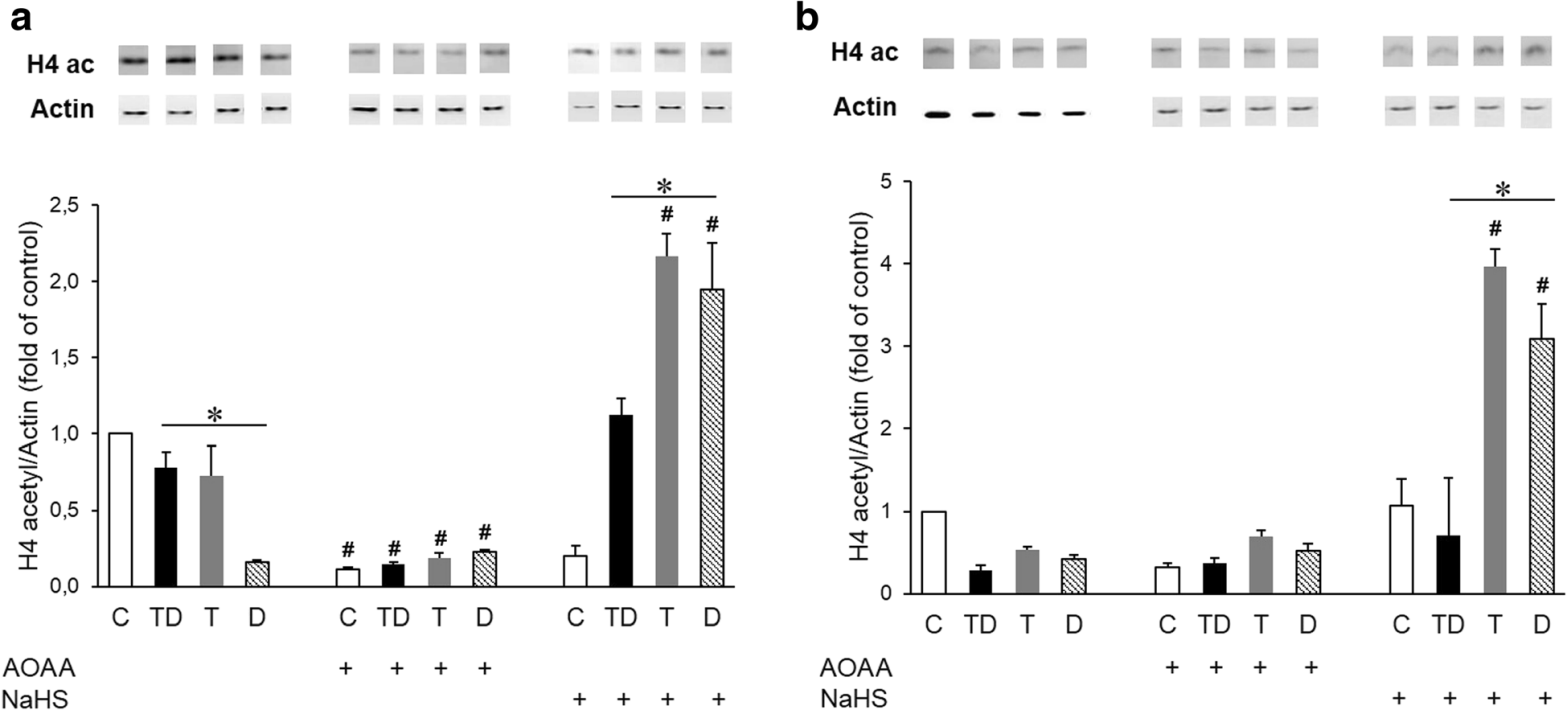
Acetylation of histone 3 is enhanced in cells treated with H_2_S. Macrophages were stimulated either with a single dose of LPS 0.5 μg/ml (T group), a single dose of LPS 0.5 μg/ml followed by a higher dose of LPS (1 μg/ml) (TD group) or directly with LPS (1 μg/ml) (D group). Histone acetylation was measured at 4 (**a**) or 24 (**b**) hours. The H_2_S biosynthesis inhibitor AOAA and the H_2_S donor were both applied at 1 mM. *p < 0.05 TD vs. D groups; #*p* < 0.05 shows the inhibitory effect of AOAA, or the stimulatory effect of NaHS, compared to the respective group that did not receive any treatment with H_2_S modulators. From each experimental group, 3 samples were loaded on the Western blot gels, and the westerns were repeated 2 times. Bar values represent the mean ± SEM of the actin-corrected densitometry values. Western blot insets above the bars show representative acetylated H3 histone and actin bands

**Fig. 7:**
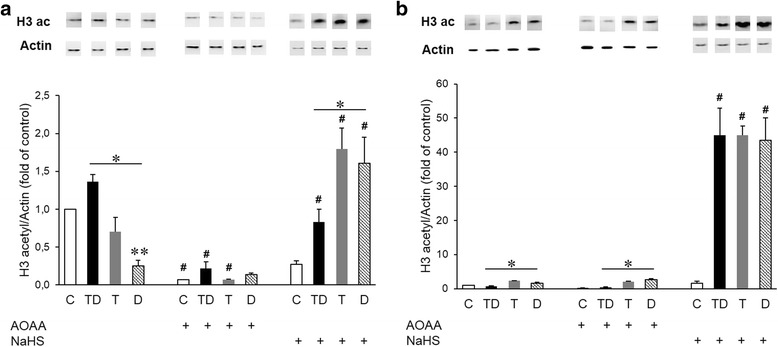
Acetylation of histone 4 is enhanced in cells treated with H_2_S. Macrophages were stimulated either with a single dose of LPS 0.5 μg/ml (T group), a single dose of LPS 0.5 μg/ml followed by a higher dose of LPS (1 μg/ml) (TD group) or directly with LPS (1 μg/ml) (D group). Histone acetylation was measured at 4 (**a**) or 24 (**b**) hours. The H_2_S biosynthesis inhibitor AOAA and the H_2_S donor were both applied at 1 mM. *p < 0.05 TD vs. D groups; #*p* < 0.05 shows the inhibitory effect of AOAA, or the stimulatory effect of NaHS, compared to the respective group that did not receive any treatment with H_2_S modulators. From each experimental group, 3 samples were loaded on the Western blot gels, and the westerns were repeated 2 times. Bar values represent the mean ± SEM of the actin-corrected densitometry values. Western blot insets above the bars show representative acetylated H4 histone and actin bands

Chromatin remodeling is a hallmark of alterations in gene expression, and changes in histone acetylation constitute a key component of this response. We have recently demonstrated that H_2_S can modulate gene expression and cytokine production through the modulation of chromatin remodeling in activated macrophages in vitro [27] Therefore, next, we tested whether the changes in expression of IL-6 and TNF-α shown in Figs. 4 and 5 are associated with chromatin remodeling, and whether H_2_S inhibition or H_2_S donation modulates these responses in the context of LPS tolerance. We observed a reduced acetylation of both histones 3 and 4 in cells exposed to the higher concentration of LPS in the shorter (4 + 4 h) LPS tolerance protocol (Fig. 6a) and of H4 in the longer (24 + 24 h) protocol (Fig. 7b). AOAA, the inhibitor of endogenous H_2_S production, reduced histone acetylation in the shorter-term (4 + 4 h) tolerance protocol in all four experimental groups, but did not affect histone acetylation in the longer-term (24 + 24 h) protocol. In contrast, treatment of the cells with the H_2_S donor increased acetylation of H3 and H4, both in the shorter and the longer-term LPS tolerance protocols, with the effect being less pronounced in the “TD” group compared to the “T” or “D” groups (Figs. 6 and 7). In summary, histone acetylation was enhanced in the presence of H_2_S donor, an effect, which partially correlated with the inhibitory effect of the H_2_S donor on the production of IL-6 and TNF-α in the same experimental protocol.

### LPS-induced production of IL-6 and TNF-α is reduced in CSE-deficient mice during endotoxin tolerance

**Fig. 8:**
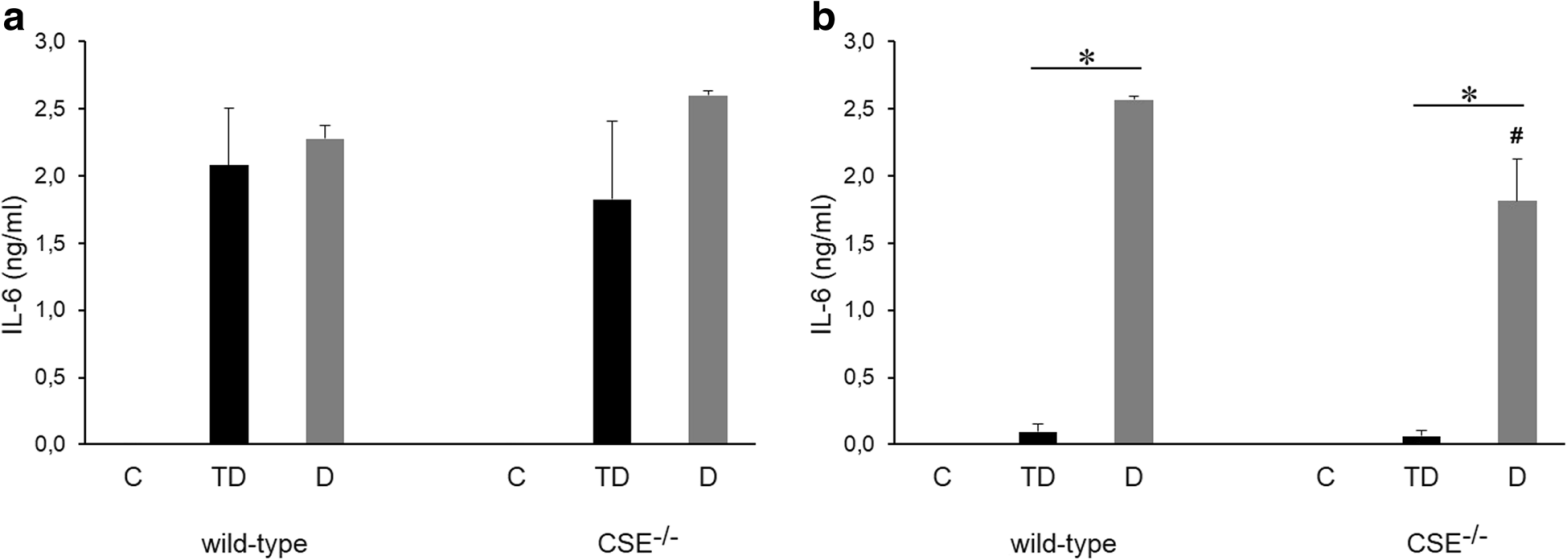
Production of IL-6 in the plasma of LPS-treated wild-type and CSE^-/-^ mice during LPS tolerance. C57/J6 black wild type and CSE^-/-^ mice were randomized into the following groups: C group: vehicle treatment; TD group: 1 mg/kg LPS during 3 days, followed by the induction of endotoxemia with 10 mg/kg LPS; D group: vehicle (instead of the tolerizing doses of LPS) before the challenge with 10 mg/kg LPS. Animals were euthanized at 4 (**a**) or 12 (**b**) hours after exposure to 10 mg/kg LPS. *p < 0.05 TD vs. D groups; #*p* < 0.05 shows the inhibitory effect of CSE deficiency, compared to the respective wild-type group

**Fig. 9:**
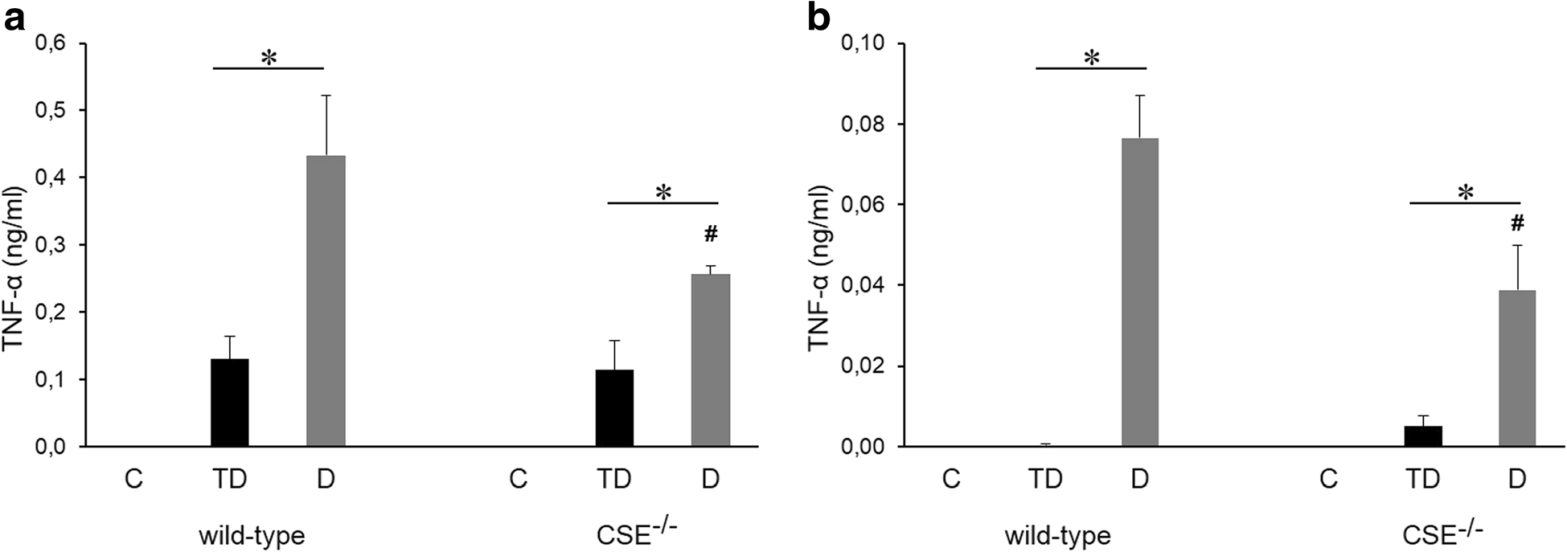
Production of TNF-α in the plasma of LPS-treated wild-type and CSE^-/-^ mice during LPS tolerance. C57/J6 black wild type and CSE^-/-^ mice were randomized into the following groups: C group: vehicle treatment; TD group: 1 mg/kg LPS during 3 days, followed by the induction of endotoxemia with 10 mg/kg LPS; D group: vehicle (instead of the tolerizing doses of LPS) before the challenge with 10 mg/kg LPS. Animals were euthanized at 4 (**a**) or 12 (**b**) hours after exposure to 10 mg/kg LPS. *p < 0.05 TD vs. D groups; #*p* < 0.05 shows the inhibitory effect of CSE deficiency, compared to the respective wild-type group

Next, we compared the production of IL-6 and TNF-α in a mouse model of LPS tolerance in wild-type (WT) and CSE knockout (CSE^-/-^) mice. To induce tolerance, animals were treated with a low dose (0.5 mg/kg/day) of LPS, followed by a single high dose (10 mg/kg) of LPS (animal group “TD”). Another group of animals was directly challenged with the high dose (10 mg/kg) of LPS (animal group “D”) without any (tolerizing) pretreatment of a lower dose of LPS. Similar to the in vitro studies, we have used shorter-term and longer-term protocols; in one protocol the time of tolerizing and challenge was both 4 h and 4 h (Figs. 8a and 9a); in another protocol, the time of tolerizing, as well as the time of high-dose LPS challenge was both 12-12 h (Figs. 8b and 9b). In the shorter-term protocol, tolerance developed with respect to TNF-α production (Fig. 9a), but - similar to our findings with the shorter in vitro LPS tolerance protocol - it did develop not with respect to IL-6 production (Fig. 8a), while in the longer-term protocol, both mediators exhibited the expected tolerance phenomenon, i.e. the LPS-induced mediator production was significantly lower in the “TD” group when compared to the “D” group (Figs. 8b and 9b). CSE deficiency failed the affect the overall pattern of these responses; LPS tolerance continued to develop in the CSE^-/-^ mice; the main difference that we have observed between wild-type and CSE^-/-^ mice was that the LPS-induced cytokine responses were less pronounced in the “TD” group than in the “D” group in the longer-term protocol (Figs. 8b and 9b) and, in the case of TNF-α production, both in the shorter-term and the longer-term protocols (Fig. 8a).

## Discussion

Gene expression programs in response to microbes requires highly precise regulatory mechanisms in innate immune system cells. LPS tolerance is a well-known phenomenon that reduces cytokine release and inflammation, where the conditioning of the genes is dependent on modification of histones, and this is associated with selective reprogramming of several genes [8–11]. It has been shown that two categories of chromatin modifications induced by tolerance: one class associated with silencing of pro-inflammatory genes and a second class, associated with antimicrobial effectors [11].

The early stimulus with lower dose of LPS initiates a complex response of “cell reprograming”, a process that involve, among many factors, epigenetic regulatory processes, including histone acetylation. Prior literature shows a promoter-specific NF-κB recruitment and histone acetylation in the context of multiple LPS-mediated pro-inflammatory and anti-microbial genes [13]. Another body of prior work suggests that LPS tolerance produces an epigenetic regulation that is locus-specific through the delimitation of the acetylation [11].

Our results confirmed that induction of LPS tolerance decreases the production of the pro-inflammatory cytokines TNF-α and IL-6 in vitro [8, 10, 27]. Our data also confirm that tolerance induction reduces cytokine production in an in vivo model of sepsis, especially in the longer-term protocol employed. We have also demonstrated that LPS tolerance is associated with marked changes in histone acetylation, although in a rather complex pattern, which does not always or directly mirror the observed changes in cytokine production. In other words, the changes in the acetylation of histones during the development of LPS tolerance (as well as in response to the subsequent high-dose LPS challenge) are dynamic and histone-specific. The most consistent pattern that was observed was that the increases in cytokine production in response LPS tend to be associated with reduced histone acetylation, and tolerance tends to increase/reverse these alterations in histone acetylation, while also suppressing cytokine production. This is also consistent with prior reports indicating that during LPS tolerance, histone acetylation contributes to the silencing of pro-inflammatory gene transcription [28–32]. Moreover, our group demonstrated an association between the reduction of cytokine release and the decrease on histone H3 acetylation at the IL-6 and TNF-α promoters in the cell exposed to H_2_S or H_2_S + LPS [27].

As far as the effect of H_2_S modulation, in the cell-based model, the most consistent and most pronounced finding was that the H_2_S donor markedly reduced pro-inflammatory cytokine production, and these effects tended to coincide with marked increases in histone acetylation. These findings continue to be consistent with the patterns seem in our in vitro experimental system, whereby - generally - higher histone acetylation corresponds to lower cytokine production, while lower histone acetylation corresponds to higher cytokine production. However, these patterns are not universally applicable. For instance, in the short-term protocol, the changes in TNF-α production do not correspond with the changes in IL-6 production, even though, obviously, the histone acetylation patterns (at least, on the macro-scale of total H3 and H4 acetylation) are the comparable. Moreover, the inhibitory effect of the CBS/CSE inhibitor AOAA on histone acetylation (although it does mirror the stimulatory effect of H_2_S donor on histone acetylation) did not manifest in any detectable change in LPS-induced cytokine production. Clearly, histone acetylation is only one of many pathways induced by LPS and/or affected by H_2_S biosynthesis modulation, and the net results (such as cytokine production and the development of tolerance) are the result of a whole host of interacting factors, only some of which have been investigated here.

When designing the current set of experiments, our working hypothesis was that H_2_S production may be a contributing factor in the development of LPS tolerance. However, the results did not support this hypothesis; the phenomenon of LPS tolerance has developed regardless whether H_2_S production was attenuated (by pretreating the cells with AOAA), or when H_2_S levels were enhanced (by pretreating the cells with the H_2_S donor NaHS). The presence of the H_2_S donor appeared to potentiate the effect of tolerance (resulting in very low cytokine levels in the “TD” group in the longer-term experimental protocols), perhaps indicative that the anti-inflammatory pathways that tolerance induces and the anti-inflammatory pathways that H_2_S induces are additive or synergistic.

However, the in vivo studies of LPS tolerance are not consistent with the conclusions made in the in vitro model: based on the effects of H_2_S in the in vitro model (where H_2_S suppresses cytokine production and enhances the anti-inflammatory effect of tolerance), we expected that CSE^-/-^ mice (that have reduced H_2_S levels) would respond with higher cytokine production or a lesser degree of tolerization; however, the data showed that tolerance developed in CSE^-/-^ mice the same way as in wild-type mice, and, in fact, the amount of cytokines produced in response to high-dose LPS was lower than in wild-type mice.

The current paper has several limitations. For instance, using CSE^-/-^ mice, one can only probe one source of H_2_S (the one produced by CSE). CSE^-/-^ mice have lower levels of circulating H_2_S, but, nevertheless, circulating H_2_S levels are still detectable [33]. Other components of the circulating H_2_S levels (i.e. H_2_S produced by cystathionine-β-synthase [CBS] or 3-mercaptopyruvate sulfurtransferase [3-MST] remain to be investigated in future studies). Another weakness of the current study is that we have only utilized one type of H_2_S source, the salt NaHS. This compound generates high levels of H_2_S in the tissue culture medium, which, then decreases over time due to a combination of cellular metabolism and physical processes (outgassing from the culture medium) [34, 35]. Follow-up studies may use H_2_S donors with longer half-life (e.g. the compound GYY4137) [35, 36] or with H_2_S donors that are targeting H_2_S to various cellular compartments (e.g. the mitochondrially targeted H_2_S donor AP39) [37].

## Conclusions

Taken together, the current findings are consistent with several lines of independent observations [8–13, 18, 28–32, 38, 39] showing or suggesting that histone acetylation is modulated during LPS tolerance development, and suggest that histone acetylation may, at least in part, contribute to LPS tolerance. The findings also confirm prior observations [27, 40–44] showing that H_2_S donors can suppress LPS-induced cytokine production in vitro. In contrast, in vivo, LPS-induced H_2_S production is *lower* in the CSE^-/-^ mice (which exhibit lower circulating levels of H_2_S) [33], suggesting that in this model - as opposed to the results of our in vitro experiments where inhibition of endogenous H_2_S production did not affect cytokine production - endogenously produced H_2_S enhances systemic cytokine production. Finally, the presence or absence of H_2_S does not appear to play a major role in the development of LPS tolerance in the in vitro and in vivo models used in the current study.

## Abbreviations

AOAA: aminooxyacetic acid
BCA: bicinchoninic acid
CBS: cystathionine-β-synthase
CSE: cystathionine-γ-lyase
ELISA: enzyme linked immunosorbent assay
ERK: extracellular-signal-regulated kinases
FBS: fetal bovine serum
H_2_S: hydrogen sulfide
IL-6: interleukin 6
LPS: lipopolysaccharide
MAP kinase: mitogen activated protein kinases
NaHS: sodium hydrosulfide
O_2_: oxygen
PDF: polyvinylidene fluoride
PMA: phorbol myristate acetate
RIPA: radioimmunoprecipitation assay
RNS: reactive nitrogen species
ROS: reactive oxygen species
SDS: sodium dodecyl sulfate
TBST: Tris-buffered saline and Tween 20
THP1: Tamm Horsfall Protein 1
TNFα: Tumor Necrosis Factor α
